# Potential biocontrol efficiency of *Trichoderma* species against oomycete pathogens

**DOI:** 10.3389/fmicb.2022.974024

**Published:** 2022-09-06

**Authors:** Yinglong Liu, Pengbo He, Pengfei He, Shahzad Munir, Ayesha Ahmed, Yixin Wu, Yuling Yang, Junping Lu, Jiansong Wang, Jizhou Yang, Xinlong Pan, Yangyang Tian, Yueqiu He

**Affiliations:** ^1^State Key Laboratory for Conservation and Utilization of Bio-Resources in Yunnan, Yunnan Agricultural University, Kunming, China; ^2^Hongta Tobacco (Group) Co. Ltd., Yuxi, China

**Keywords:** oomycete disease, biological control, *Trichoderma* spp., control effect, antagonisim

## Abstract

Plant health is of utmost importance for optimal agricultural production and sustainability. Unfortunately, biotic and abiotic factors put a major constraint on crop safety and productivity. Plant diseases caused by oomycetes inflict serious damage to various crops. Moreover, the injudicious use of chemical pesticides poses threats related to pesticide resistance development in pathogens and environmental pollution. Biocontrol offers an effective solution for disease control; however, research on biocontrol of oomycete-related diseases is scarce. Thus, this study undertakes the screening of biocontrol resources for the effective management of oomycete-related plant diseases. In this regard, 86 isolates of *Trichoderma* spp. were assessed against *Phytophthora nicotianae, P. capsici, Pythium vexans, P. ultimum*, and *P. dissotocum* through dual culture assay. Furthermore, the antagonistic effect of selected isolates was studied against tobacco black shank disease and damping-off of cucumber seedlings in the greenhouse. The relative control effect of the three antagonistic *Trichoderma* strains AR-4, Tv-1, and ST4-1 on tobacco black shank was more than 60%, which was not significantly different from 6.88 gl^−1^ fluopicolide–propamocarb. Whereas, the relative control effect of *Trichoderma* AR-4 and ST4-1 on damping-off of cucumber seedlings was 80.33% and 82.67%, respectively, which were significantly higher than *Trichoderma* Tv-1 (35.49%) and fluopicolide–propamocarb (47.82%). According to the morphological and molecular characterization, the fungal strains AR-4, Tv-1, and ST4-1 were identified as *Trichoderma koningiopsis, T. asperellum*, and *T. gamsii*, respectively. In conclusion, the strains exhibited a strong antagonistic effect against oomycete pathogens and can be integrated into disease management strategies.

## Introduction

Agricultural production is dependent on the optimal functioning and health of plant. Unfortunately, plant disease is a major restricting factor in crop yield and quality (Ahmed et al., 2020). Oomycetes pose a serious threat to agriculture and food production, cause devastating diseases in hundreds of plant species and severely limit agricultural development (Judelson and Blanco, 2005). Common oomycetes cause root rot, fruit rot, dieback, leaf spot, and stem canker of horticultural plants and crops (https://gd.eppo.int/; https://Phytophthora.ucr.edu/; World Phytophthora Genetic Resource Collection). For example, *Phytophthora infestans* caused the Irish potato famine in the 1840s. Whereas, *P. cinnamomi* is the most invasive species also known as the biological bulldozer and *P. capsici* is a highly destructive pathogen. Moreover, tobacco black shank (TBS) disease caused by *P. nicotianae* is the most prevalent and destructive soil-borne disease in cultivated tobacco worldwide (Kamoun et al., 2015). Similar to *Phytophthora* spp., it is known that *Pythium* spp. is also a group of pathogenic oomycetes which cause several common plant diseases. It survives in soil and on plant residues as a saprophyte and produces pectinase and cellulase to promote the degradation of plant cell wall where mycelia gain entry into the plant and cause plant root rot, damping-off of seedlings, and crop dysplasia (Zerillo et al., 2013; Reeves et al., 2021). Another common pathogen, *Pythium vexans* is widely distributed in plant rhizosphere and environmental soil and causes root rot in cucumber, tomato and other vegetables and a variety of crops, seriously limiting crop production (Galland and Paul, 2001). In addition, *P. ultimum* can cause humidification and root rot on more than 300 species of crops, trees, and horticultural plants and is listed as one of the top 10 pathogens of oomycetes (Kamoun et al., 2015). At present, the most effective method to control oomycete diseases is still based on chemical agents (Leadbeater, 2015). However, the loss of multi-gene resistance in resistant varieties or the continuous application of a single chemical agent leads to genetic variations and development of pesticide resistance in pathogens. Chemical pesticides are also deleterious toward environmental and public health (Bardin et al., 2015; Parween et al., 2016). Therefore, it is urgent to find an effective and eco-friendly solution to prevent or control plant diseases.

Environment protection and food security have gained much recognition around the globe. Therefore, non-chemical and environment-friendly disease control technologies based on biocontrol are now being widely adopted. At present, the biological control of plant diseases mainly relies on beneficial microorganisms and microbial metabolites. Beneficial microorganisms include bacteria, actinobacteria, fungi, and viruses, which are closely associated with plant health and safety (Munir et al., 2021; Ahmed et al., 2022). Microbial metabolites, such as validamycin and streptomycin, are the most widely used biological pesticides at present (Copping and Menn, 2000; Bian et al., 2021). *Trichoderma* was first isolated from soil in 1794 and it has been a well-known biocontrol resource since then. *Trichoderma* is considered an efficient biocontrol agent due to low development cost, strong adaptability, broad-spectrum efficiency and ecological friendliness, and broad application prospects (Sood et al., 2020). The biocontrol mechanism mainly includes the synthesis of specific compounds and mobilization of certain nutrients to plants that promote plant growth and improve defense (Bononi et al., 2020). The biocontrol agents eliminate pathogenic microbes through niche and nutrient competition as they efficiently colonize plant rhizosphere and plant parts (Sood et al., 2020). They also strengthen plant immunity against pathogens by the induction of resistance in the host (Adnan et al., 2019). They produce a wide range of metabolites that either directly target the pathogen or act as a signal of the activation of the plant defense system. *Trichoderma* is also well documented for parasitizing pathogens (Vos et al., 2015; Li et al., 2019; Khan et al., 2020; Lazazzara et al., 2021).

Introduction of new biocontrol strains in the form of *Trichoderma* has several benefits to act as a biostimulant and plant growth promoters. However, weather variability also needs to be considered in long run for the management of emerging pathogens recently (Kovács et al., 2021). New biocontrol agents should have potential effectiveness to inhibit the pathogen under varying weather and climatic conditions (Modrzewska et al., 2022). The biocontrol strain inoculation before or after pathogen attack and delivery method plays an important role to understand the preventive effect. The beneficial biocontrol strain should not only inhibit the pathogen growth but also need to improve plant growth. A newly identified novel fungus, *Trichoderma phayaoense*, was reported not only for plant growth promotion but also for inhibiting the pathogen of gummy stem blight and wilt of muskmelon (Nuangmek et al., 2021). However, colonization efficiency *Trichoderma* spp is also important to contribute to disease-suppressive abilities in different soils and adherences to the root system (Poveda, 2021b). As far as nematodes are concerned, most of the *Trichoderma* strains are used against the *Meloidogyne* spp. in different crops (Jindapunnapat et al., 2013). Interestingly, several studies focus on the use of this genus for plant survival in extreme drought and temperature. The strain of *T. harzianum* was used in tomato plants to reduce abiotic stress (Mastouri et al., 2010; Ahmad et al., 2015).

The current research undertakes the screening of *Trichoderma* spp. with antagonistic activity against common pathogens of oomycete origin. The control effect of selected strains against TBS disease and damping-off of cucumber seedlings was further assessed in the greenhouse. The study lays a theoretical foundation to incorporate *Trichoderma* in the disease management of oomycetes.

## Materials and methods

### Strains and cultural conditions

In total, 86 *Trichoderma* strains were isolated from plant's (apple, pear, tobacco, cucumber, *Panax notoginseng*) stems, roots, and rhizosphere soil, and were preserved by filter paper method in the Molecular Plant Pathology Lab, Yunnan Agricultural University, Yunnan Agricultural University, Yunnan Province, China. Afterward, the isolates were inoculated on the potato dextrose agar (PDA) medium and incubated at 28°C for further use.

The oomycete pathogens: *Phytophthora nicotianae, P. capsici, Pythium vexans, P. ultimum*, and *P. dissotocum* were isolated from related diseased plants, identified through morphological and molecular characterization as performed in our previous study (Liu et al., 2022) and stored in our lab as mentioned above. For further experiments, the isolates were inoculated on the oatmeal agar (OA) medium and incubated at 28°C.

The lethality of *Phytophthora* spp. or *Pythium* spp. was determined on oomycetes selective medium, composed of oat medium, and supplemented with fungicide and antibiotics (each liter of oat medium contained rifampicin 25–75 mg, ampicillin 50–100 mg, nystatin 25–75 mg, hymexazol 50–100 mg, pentachloronitrobenzene 75–150 mg, and thiophanate-methyl–tebuconazole 25–75 mg, pH value is 6.8–7.2), the incubation conditions were the same as mentioned above.

### Antagonistic effect of *Trichoderma* on oomycete pathogens

In order to assess the antagonistic effect of *Trichoderma* against oomycete pathogens (*P. nicotianae, P. capsici, P. vexans, P. ultimum*, and *P. dissotocum*), dual culture assay was opted. First, oomycete pathogens were cultured on the OA medium, and *Trichoderma* isolates were cultured on the PDA medium for 3–7 days. Afterward, pathogen and *Trichoderma* colonies were perforated with a sterilized 7 mm perforator, respectively, and were removed with the sterile needles and placed upside down at both ends of the PDA plate with a diameter of 9 cm. The plates were kept in dark at 28°C for 7 days and observed whether *Trichoderma* hyphae could cover or antagonize the pathogen colonies. Each treatment consisted of six plates. Finally, the growth diameter of *Trichoderma* and pathogens was measured, and the relative inhibition rate of *Trichoderma* against pathogens was calculated using the following formula (El-Debaiky, 2017).


$$\begin{array}{l} \text{Relative inhibition rate }\left(\%\right)\\ =\frac{\left(\text{Colony diameter of control }-\text{ Colony diameter of treatment}\right)}{\text{Colony diameter of control}}\\ \times 100 \end{array}$$

### Determination of the lethality of *Trichoderma* toward oomycete pathogens

The lethality of *Trichoderma* toward oomycete pathogens in the inhibition area was further studied. In this regard, plates showing significant antagonistic effects were selected, and three mycelium discs of the pathogen from 7 to 10-days-old plates of the dual culture of *Trichoderma* and pathogen were extracted with a sterilized 7 mm perforator. The discs were transferred to the selective medium plate with a sterile pick needle, and incubated in darkness at 28°C for 3–7 days, while pure cultures of *Phytophthora* spp. or *Pythium* spp. served as controls. Afterward, the plates were observed for the growth of Phytophthora spp. or Pythium spp. mycelium on selective medium and the experiment was repeated thrice.

### Control effect of TBS disease and damping-off of cucumber seedlings

TBS disease caused by *P. nicotianae* and damping-off of cucumber seedlings caused by *P. vexans* were used as the model diseases, and healthy plants were inoculated with pathogens and *Trichoderma* to determine the control effects of selected *Trichoderma* isolates against these pathogens. Briefly, soil was sterilized and seeds of tobacco variety KRK26 were sown in the floating system, while the seeds of cucumber variety Xintangshan Qiugua were sown in small pots (12 × 13 cm) with more than five seeds per pot. After the tobacco seedlings reached to the four leave stage and cucumber seeds grew true leaves, the tobacco seedlings were transplanted to the same size pots for a week and selected the cucumber seedlings with consistent emergence. Meanwhile, spore suspensions of pre-cultured pathogens and *Trichoderma* were prepared. The plates covered with mycelia of *P. nicotianae* or *P. vexans* were soaked in 0.1% potassium nitrate solution for 3 days to induce sporulation (Galiana et al., 2019). Sterile water was added to the plates covered with mycelia of Trichoderma and the plates were repeatedly and repeatedly scraped with an applicator, and after filtering, the concentration was adjusted to 1 × 10^6^ spores·ml^−1^ with sterile water. In total, 50 ml of pathogen spore suspension per plant/pot was applied to the roots of tobacco and cucumber seedlings, respectively. The next day, 50 ml of *Trichoderma* spore suspension was applied to the roots, and 50 ml of 687.5 gl^−1^ fluopicolide-propamocarb (SC, suspension concentrate, Bayer Crop Science (China) Co., Ltd.) 100-fold dilution and sterile water served as a positive and negative control, respectively, and each treatment consisted of eight plants per pot. The experiment was repeated three times. On the 1st, 8th, 15th, and 22nd day after treatment, the disease index of TBS disease (Guo et al., 2020), the incidence of damping-off of cucumber seedlings, and their relative control effect were recorded using the following formulae (Hossain et al., 2014; Huang et al., 2020).


$$\begin{array}{l} \text{Disease index}=\frac{\sum \left(\text{Number of diseased plants or leaves at each level }\times \text{ The disease grade value}\right)}{\text{The total number of investigated plants or leaves }\times \text{ The highest value}}\times 100\\ \text{Incidence rate}\left(\%\right)=\frac{\text{Total number of diseased plants }}{\text{The total investigated number of plants}}\times 100\\ \text{Relative control effect}\left(\%\right)=\frac{\left(\text{Disease index of control}-\text{Disease index of treatment }\right)}{\text{Disease index of control}}\times 100 \end{array}$$

or:


$$\begin{array}{l} \text{Relative control effect }\left(\text{\%}\right)=\frac{\left(\text{Incidence rate of control}-\text{Incidence rate of treatment}\right)}{\text{Incidence rate of control}}\times 100 \end{array}$$

### Morphological characteristics and molecular biological identification of *Trichoderma* isolates

*Trichoderma* isolates with strong biocontrol potential were characterized through morphological and molecular parameters. First, *Trichoderma* isolates were cultured on the PDA plate and their appearance, morphology, and colony color were recorded. Their mycelia, conidiophores, conidia, and chlamydospores were observed and photographed by optical microscope (Carl Zeiss Microlmaging GmbH 37081 Gottingen, Germany). Furthermore, for molecular characterization, the genomic DNA of *Trichoderma* isolates was extracted by the CTAB method (Munir et al., 2020), and subsequently, the internal transcribed spacer (ITS) of ribosome sequence was amplified by primers ITS1 (5′-TCCGTAGGTGAACCTGCGG-3′)/ITS4 (5′-TCCTCGCTTATTGATATGC-3′) (White et al., 1990) and the PCR products were sequenced from Shanghai Qingke Biotechnology Co., Ltd. Obtained sequences were aligned with the sequences from GenBank (http://www.ncbi.nlm.nih.gov/blast/), and the homology of sequences was analyzed using MEGA 6, and the phylogenetic tree was constructed by the neighbor-joining method.

### Statistical analysis

For the statistical analysis, SPSS24.0 was used to calculate the average value and analyze the variance of the test data, while Duncan's new multiple range test was applied for multiple comparisons (*P* ≤ 0.05).

## Results

### Antagonism and inhibition rate of *Trichoderma* on pathogens

**Figure 1:**
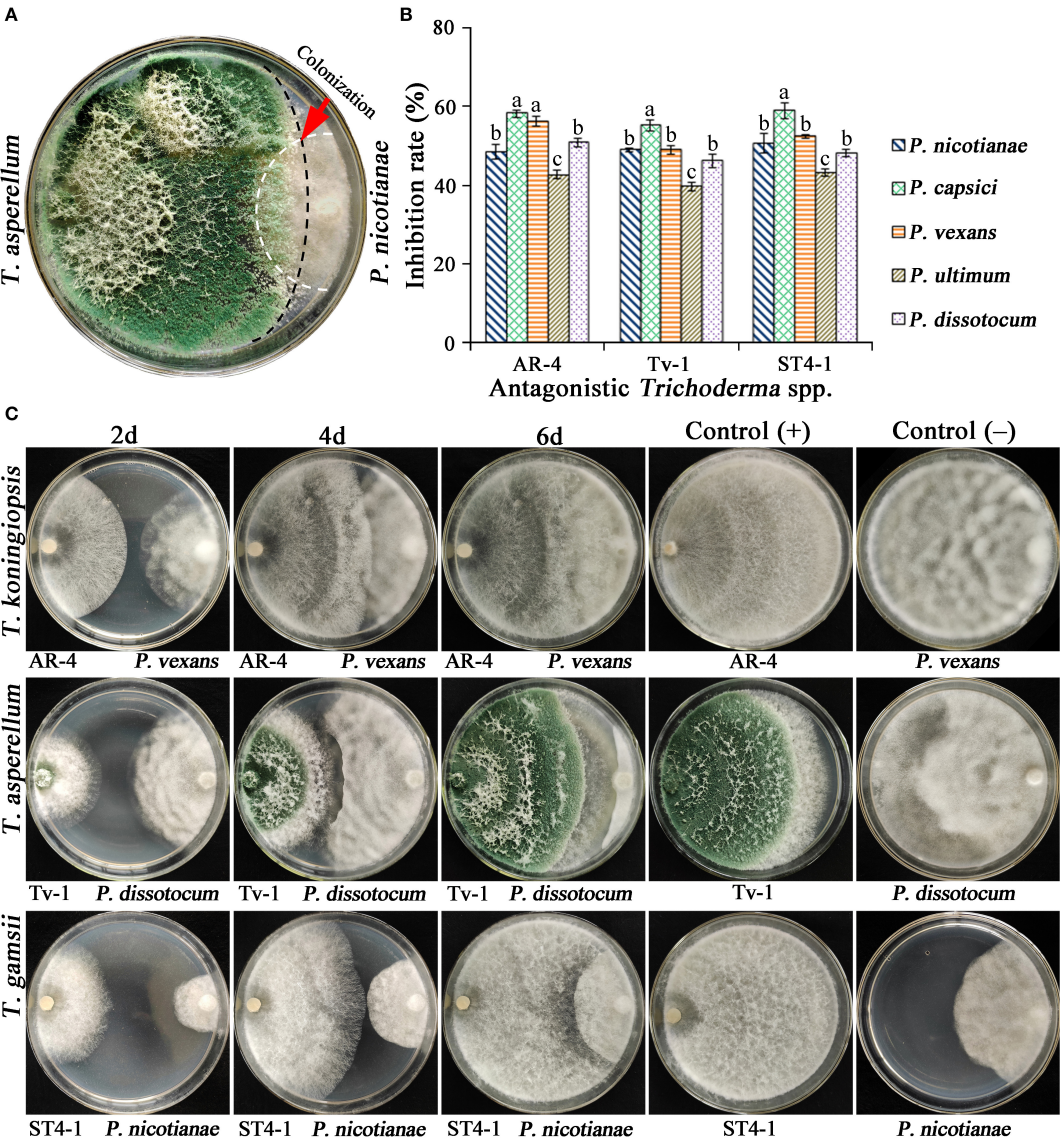
Antagonism of three antagonistic *Trichoderma* spp. against oomycete pathogens and inhibition rate. **(A)** Antagonism of *Trichoderma*, the red arrow shows the antagonistic zone of *T. asperellum* Tv-1 on *P. nicotianae* (5d); **(B)** the relative inhibition rate of three antagonistic *Trichoderma* spp. on oomycete pathogens; **(C)** antagonism of three *Trichoderma* spp. against oomycete pathogens.

Antagonistic activity of *Trichoderma* isolates on oomycete pathogens was assessed through a dual culture assay. Those isolates that were not antagonistic toward pathogens, could not grow in a large area on plates. However, *Trichoderma* showing the obvious antagonistic effect on pathogens could rapidly expand and colonize on the mycelium of pathogens (Figure 1A), whereas the mycelia of the pathogen stopped growing and the colony stopped expanding, while the *Trichoderma* continued to grow forward until it completely covered the pathogen colony and the entire plate (Figure 1C). In addition, some isolates only showed weak antagonistic activity on certain pathogens. Therefore, only a few isolates could antagonize all five pathogens. It was found that *Trichoderma* AR-4, Tv-1, and ST4-1 performed better than other isolates, exhibited higher growth rate and stronger antagonism and the inhibition rate on all oomycete pathogens was over 40%, indicating significant antagonism (Figures 1B,C).

### The lethality of *Trichoderma* toward oomycete pathogens

**Figure 2:**
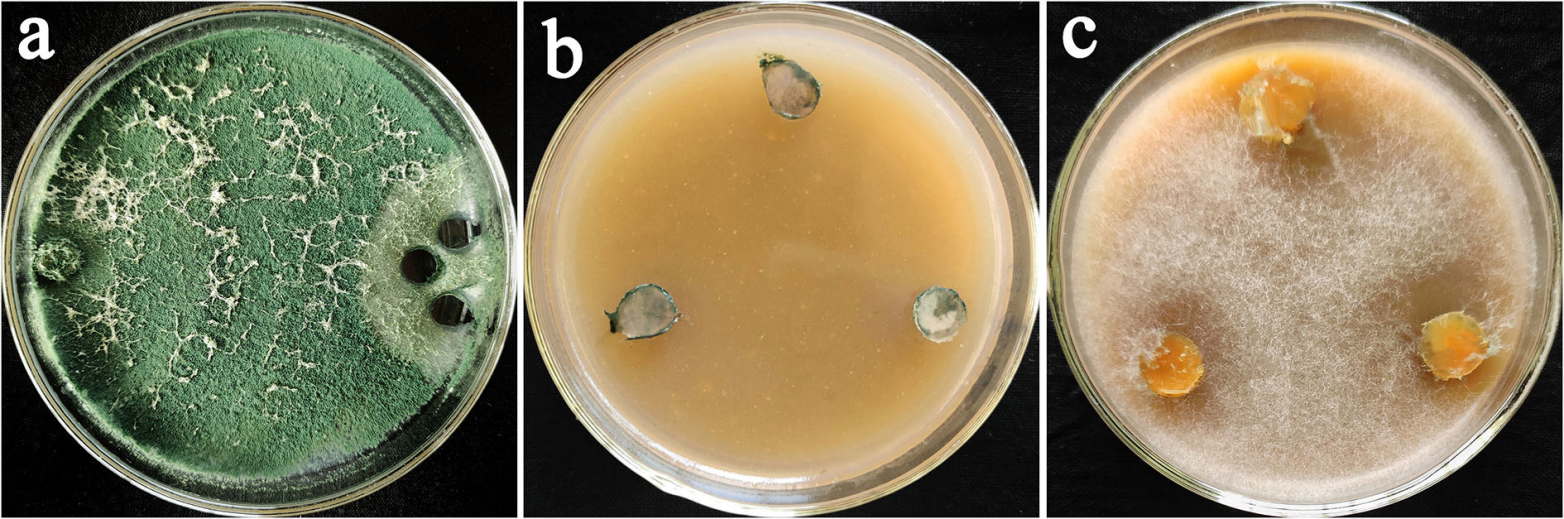
*Trichoderma* antagonized and killed oomycetes. **(a)** Antagonism of *T. asperellum* Tv-1 on *P. nicotianae*; **(b)** *P. nicotianae* could not form colonies on the selective medium after being parasitized by Tv-1; **(c)** pure *P. nicotianae* grew into colonies on the selective medium.

The oomycete pathogens antagonized by *Trichoderma* spp. in dual culture assay were re-isolated on the oomycete selective medium (Figure 2a). After continuous observation for 7 days, no colonies of *Phytophthora* spp. or *Pythium* spp. grow on selective medium plates (Figure 2b), while the single colony of *Phytophthora* spp. or *Pythium* spp. grow with white mycelium and expanded outward the mycelium disc (Figure 2c). Some *Phytophthora* spp. or *Pythium* spp. antagonized by *Trichoderma* spp. could not grow into colonies when transferred to the selective medium, which proved that *Trichoderma* spp. could not only antagonize *Phytophthora* spp. or *Pythium* spp. but also caused their inhibition death completely. Finally, three strains of *Trichoderma* spp. AR-4, ST4-1, and Tv-1 showing higher antagonism and lethality were selected for further experiments.

### Control effect on TBS disease and damping-off of cucumber seedlings

**Figure 3:**
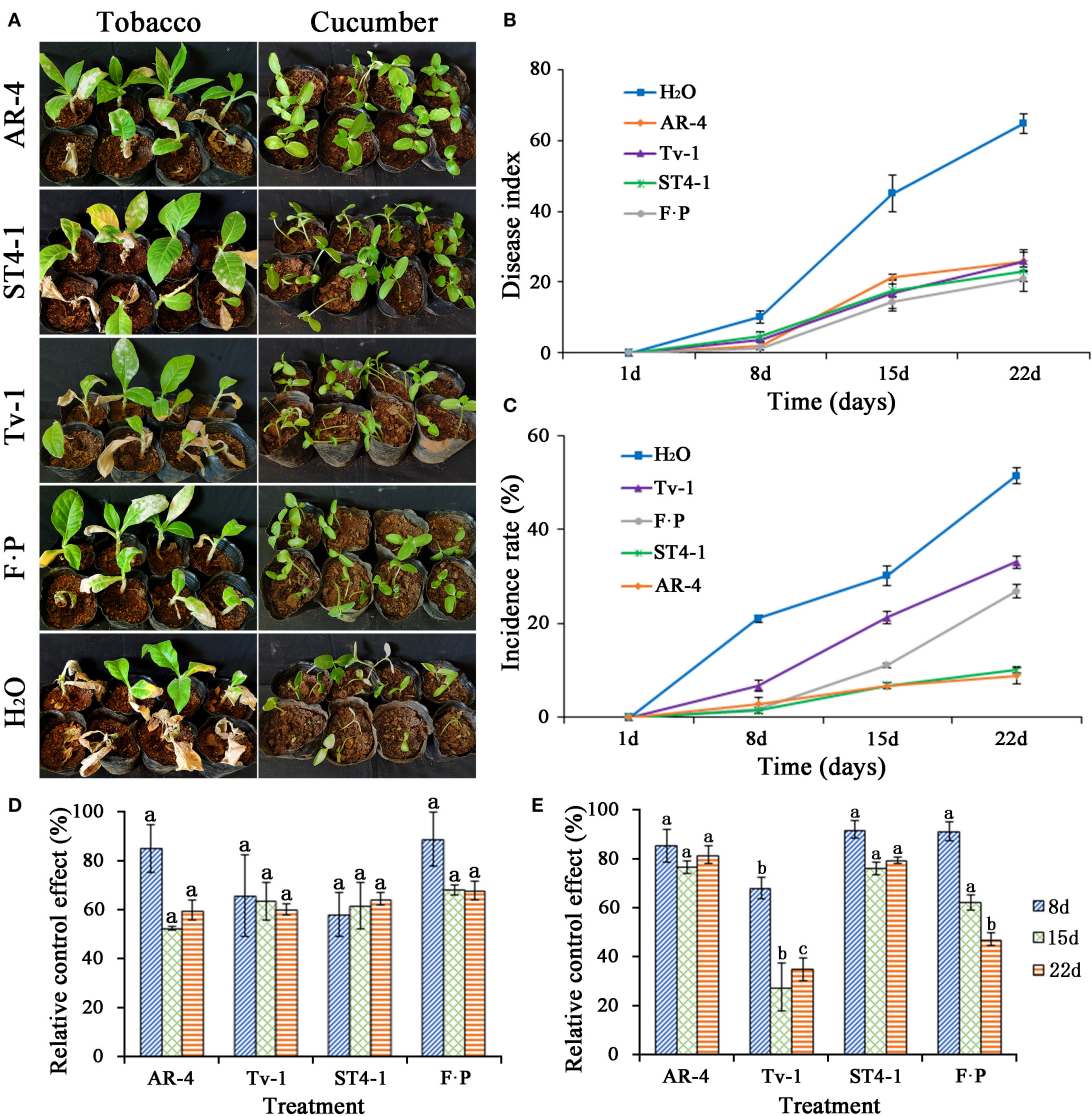
Biocontrol effect of *Trichoderma* spp. on TBS disease and damping-off of cucumber seedlings. **(A)** Disease status of tobacco and cucumber seedlings after the application of *Trichoderma* spp. and control group; **(B)** disease index of TBS disease after the application of *Trichoderma* spp. and control group; **(C)** the incidence rate of damping-off of cucumber seedlings after the application of *Trichoderma* spp. and control group; **(D)** relative control effect of *Trichoderma* spp. and fluopicolide-propamocarb (FP) on TBS disease compared with water treatment; **(E)** relative control effect of *Trichoderma* spp. and fluopicolide-propamocarb (FP) on damping-off of cucumber seedlings compared with water treatment.

The control effect of *Trichoderma* strains AR-4, ST4-1, and Tv-1 on TBS disease had no significant difference compared with the 100 times dilution of 687.5 gl^−1^ fluopicolide-propamocarb (SC). The health status of tobacco seedlings also improved, and the disease index at 22 days ranged from 20.83 to 25.93, which were significantly lower than that of the water control (disease index is 64.81) (Figures 3A,B). The results highlight that *Trichoderma* strains AR-4, ST4-1, and Tv-1 have a strong control effect on TBS disease. Compared with water control, the relative control effect in 8–22 days was more than 60.00%, which is not significantly different from the 100 times dilution of 687.5 gl^−1^ fluopicolide-propamocarb (SC) (Figure 3D). In case of cucumber seedling damping-off, the disease incidence recorded at 22 days was 51.41, 33.02, and 26.80%, for water control, *Trichoderma* Tv-1 treatment, and fluopicolide-propamocarb, respectively. Whereas, the disease incidences for *Trichoderma* strains ST4-1 and AR-4 treatments were only 10.10% and 8.82%, which was significantly lower than water treated control group (Figure 3C), and the control efficiency was 80.33% and 82.67% compared with the negative control group, significantly higher than *Trichoderma* Tv-1 (35.49%) and positive control (47.82%) (Figure 3E). The incidence of damping-off of cucumber seedlings was much lower, and health status of plants was improved (Figure 3A).

### Morphological characteristics and molecular identification of antagonistic *Trichoderma* spp.

**Figure 4:**
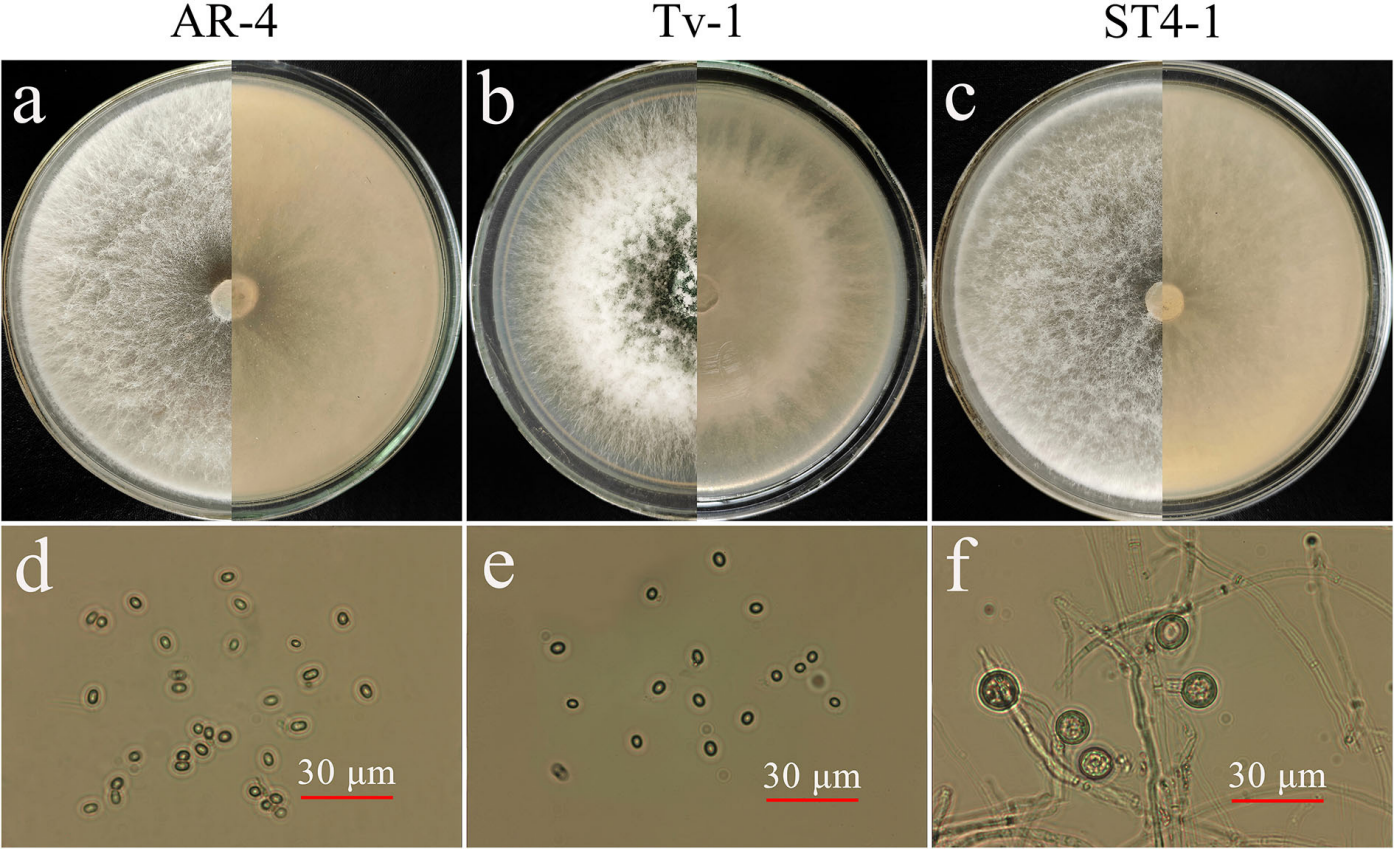
Morphological characteristics of antagonistic *Trichoderma* spp. **(a)** Colony morphology of AR-4 (5d); **(b)** colony morphology of Tv-1 (5d); **(c)** colony morphology of ST4-1 (5d); **(d)** conidia of AR-4; **(e)** conidia of Tv-1; **(f)** chlamydospores of ST4-1.

The three strains of *Trichoderma* efficiently grew on the PDA plate and forms aerial hyphae (Figures 4a,c). AR-4 colony are white and radial, turned into light green in the later stage of culture, and form a large number of conidia. The conidia are ellipsoid or spherical, light green, and diameter ranged from 8 to 15 μm (Figures 4a,d). The Tv-1 colony was white at the beginning and slowly turned green 24 hours later and dark green at the later stage of culture. The primary hyphae were cashmere like and relatively robust and well developed. In the later stage of culture, a large number of green ellipsoidal or spherical conidia layers adhered to the surface of the colony, resulting in a powdery appearance, and only a small number of aerial hyphae on the surface was arachnoid (Figures 4b,e). The mycelium of ST4-1 was fluffy and the colony appeared white, and dense felt-like colonies formed in the later stage of culture. The mycelium and conidiophore were frequently branched and intertwined with each other, with flask-shaped conidiophore. There were few conidia with a diameter of about 3–6 μm, but chlamydospores were abundant and spheroidal, with a diameter of 10–15 μm (Figures 4c,f). In addition, the strain also produced aroma such as coconut.

**Figure 5:**
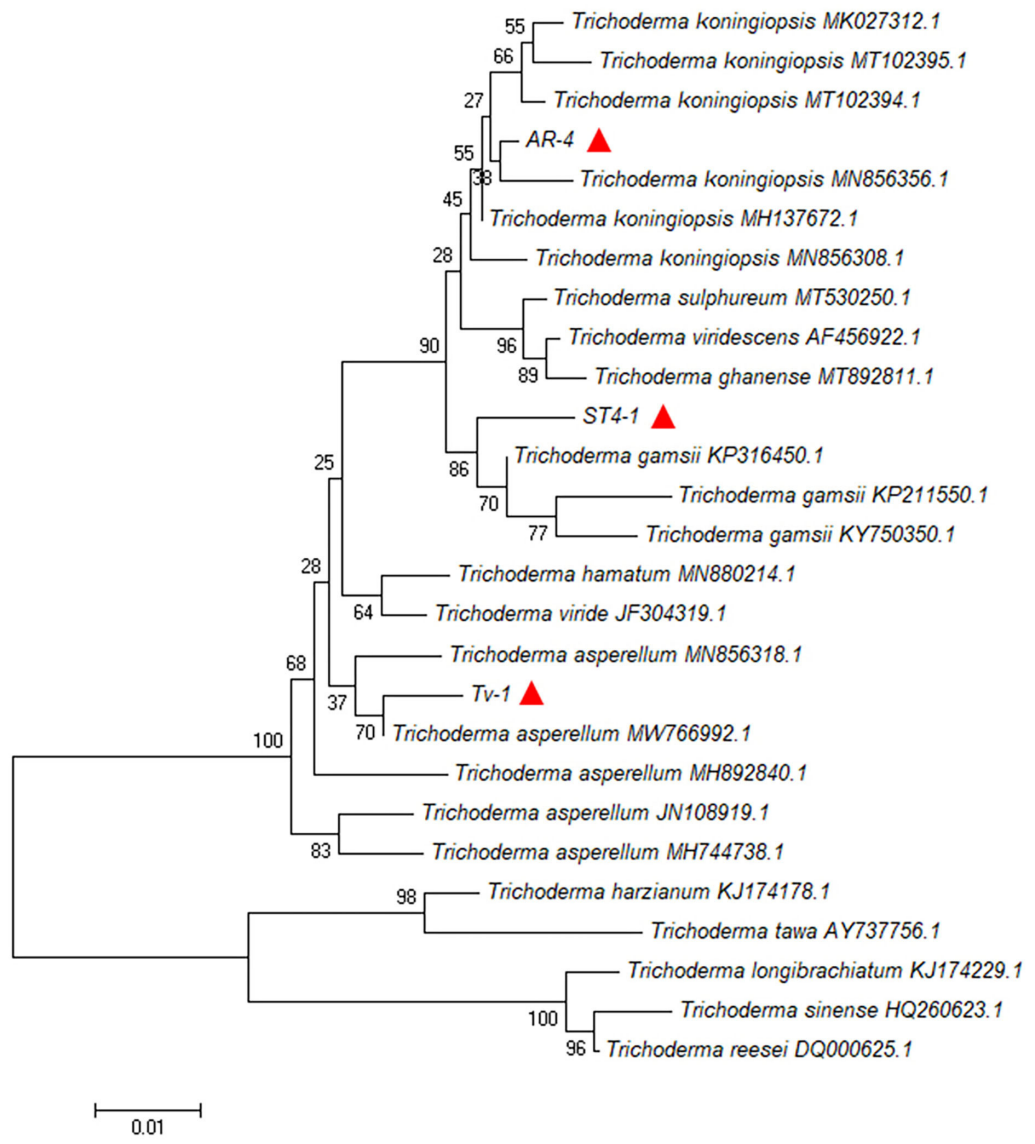
Phylogenetic tree of antagonistic *Trichoderma* strains AR-4, Tv-1, and ST4-1 based on their ITS sequences (red triangles).

The genomic DNA of the above three antagonistic *Trichoderma* strains was amplified by PCR to obtain products with a size of about 600 bp. The results of sequencing and BLAST homology comparison revealed that strains AR-4, Tv-1, and ST4-1 were closely related to *Trichoderma koningiopsis, T. asperellum*, and *T. gamsii* in the GenBank database, respectively, with sequence consistency of more than 98%. The phylogenetic tree also confirmed that the tested strains were closely related to them (Figure 5). According to the above results, strain AR-4 was identified as *T. koningiopsis*, Tv-1 was identified as *T. asperellum*, and ST4-1 was identified as *T. gamsii*. The above sequencing results have been submitted to the NCBI GenBank database, and their sequence accession numbers obtained are MZ778859 (AR-4), MZ771300 (Tv-1), and MZ778861 (ST4-1).

## Discussion

In the co-evolution of pathogen–host interaction, pathogens have evolved strategies to interfere, avoid, or weaken the host's defense response and normal physiological processes, thus creating a suitable environment that allows pathogens to grow and cause diseases (Chisholm et al., 2006). RXLR effectors are the key toxicity factors of oomycetes. During host infection, RXLR effectors extensively regulate the host response process, weaken host immunity, enter plant cells to enhance their toxicity, and accelerate plant cell apoptosis (Ren et al., 2019; Naveed et al., 2020). In addition, oomycetes can secrete a variety of enzymes, such as glycoside hydrolase (GHs), cell wall degrading enzyme (CWDEs), and pectinase, which accelerate tissue necrosis and provide pathogen entry into the plant (Yuan et al., 2021; Kuhn et al., 2022). Therefore, plant diseases caused by oomycete pathogens are common, rapidly spreading, destructive, and difficult to control.

According to the pesticide information network (http://www.chinapesticide.org.cn/hysj/index.jhtml) statistics in China, more than 99% of the registered pesticides for the prevention and control of oomycete plant diseases are chemical fungicides. For example, among 1,659 registered fungicides for the downy mildew control, only three *Trichoderma* products and two *Bacillus* products are registered, while only five *Bacillus* strains, two *Trichoderma* strains, and one *Pythium oligandrum* product among 849 are registered fungicides against *Phytophthora* spp. The long-term excessive use of chemical pesticides for plant disease control and insect pests may lead to a vicious cycle of ecological imbalance. Whereas, biological control is an environment-friendly option and should be adopted widely. Therefore, it is necessary to screen more biocontrol resources against plant pathogens and expedite the development and application of biocontrol agents.

*Trichoderma* is considered the representative of beneficial fungi. The members of this genus are a great source of enzymes, antibiotics, plant growth promoters, soil remediation agents, and commercial biological fungicides, while some of them have important scientific research value and economic significance in the fields of agriculture, medicine, and biotechnology (Daniel and Filho, 2007; Brotman et al., 2010; Oszust et al., 2021). For agricultural production, *Trichoderma* is often used as bio-organic fertilizer and fungicide, which has a positive impact on host plants. *Trichoderma* is famous for its strong ability to antagonize pathogens through a variety of strategies. Among them, antagonizing on the pathogen is the most direct interaction between biocontrol agent and pathogen. Therefore, in this study, the antagonistic effect of *Trichoderma* on oomycete pathogens has been taken as the primary screening index to select the isolate with a broad-spectrum antagonistic effect and to exclude the strains which are non-antagonistic or antagonistic toward a single oomycete. Among 86 isolates of *Trichoderma* tested, 54 isolates exhibited the antagonistic effect on the oomycete pathogen, which indicates that the antagonistic effect on the pathogen is the effective mechanism of *Trichoderma* against oomycetes. Further, 16 *Trichoderma* isolates were found to possess broad-spectrum antagonistic ability against five pathogenic oomycetes as they were able to limit the colony expansion of pathogen. The second screening index of this study was to use the specific medium for oomycete pathogen to observe whether *Trichoderma* completely antagonized oomycete or not. This method was used to further screen *Trichoderma* with lethal ability after direct action on oomycetes. In this study, not all 16 *Trichoderma* strains with broad-spectrum antagonistic ability showed lethal ability, and oomycete antagonized by seven isolates of *Trichoderma* were unable to grow on the selective medium. Therefore, only seven *Trichoderma* isolates could perform dual functions of broad-spectrum antagonism and wide inhibition. This screening strategy established in this study can efficiently screen effective isolates of *Trichoderma* in a short time and at less cost. It is a simplified and innovative screening process and lays a foundation for the biological prevention and control of oomycete plant diseases.

After screening and obtaining *Trichoderma* with the dual functions of broad-spectrum antagonism and extensive lethality, the control efficiency of oomycete plant diseases was assessed. The results showed that three antagonistic *Trichoderma* strains could stably exist in the soil and the relative control efficacy on TBS disease exceeded 60.00% at 22 days. The relative control effect of *T. koningiopsis* AR-4 and *T. gamsii* ST4-1 on damping-off of cucumber seedlings disease reached 80.33% and 82.67%, respectively. It was significantly higher than that of *T. asperellum* Tv-1 (35.49%) and 687.5 gl^−1^ fluopicolide-propamocarb (SC) (47.82%). *T. koningii* inoculated with the fungal pathogens *Fusarium oxysporum* f. sp. *cicero* results in the expression of defense-related genes, especially an increase in the expression of salicylic acid genes (Poveda, 2021a). The direct biocontrol activities of most of the *Trichoderma* spp. are due to the production of secondary metabolites, volatiles, and chitinase enzymes that can easily degrade the cell wall of pathogenic fungi (Zheng et al., 2021; Mukherjee et al., 2022). Based on these mechanisms, we showed that the selected antagonistic *Trichoderma* strains screened in this study have strong disease control ability and great potential for biocontrol application, especially against oomycete pathogens.

As an opportunist, *Trichoderma* have great survival potential and can quickly adapt to new ecological niches (Druzhinina et al., 2011). In plant protection and disease control, the identification of a biocontrol agent is based on competition and antagonism with pathogens (Reithner et al., 2011). In addition, *Trichoderma* can colonize on the surface of plant roots and penetrate epidermal cells, produce various compounds, cause substantial changes in plant proteome and metabolome, induce local or systemic resistance response, and protect against a variety of plant pathogens. At the same time, *Trichoderma* colonization on plant roots enhances the growth and development of roots and is conducive to the absorption and utilization of nutrients (Harman et al., 2004).

## Conclusion

Emerging pathogens need new biocontrol strains to control them successfully without showing adverse effects on host plants. Based on overall findings, we suggest that the selected three antagonistic *Trichoderma* strains in this study not only showed broad-spectrum antagonistic ability and lethal effects *in vitro* but also reduced the disease incidence of TBS and damping-off of cucumber seedlings *in vivo*. Despite this progress, more research is still needed to overcome limitations and improve the performance of biocontrol strains against emerging pathogens belonging to oomycetes. Finally, we found that the treated plants also exhibited enhanced growth and vigor, which warrants further research to reveal the biological mechanism operated for such plant-benefiting functions.

## Data availability statement

The original contributions presented in the study are included in the article/supplementary material, further inquiries can be directed to the corresponding author/s.

## Author contributions

YL, PengbH, PengfH, SM, YY, JL, JW, JY, and XP performed laboratory experiments and data analyses. YL, YW, YH, JL, JW, JY, and YT performed field experiments and collected samples. YT and YH supervised the study. YL, SM, AA, and YH drafted the manuscript. All authors contributed to the article and approved the submitted version.

## Funding

This study was supported financially by Hongta Tobacco (Group) Co. Ltd., Yuxi (2020YL04).

## Conflict of interest

Authors JL, JW, JY, and YT were employed by Hongta Tobacco (Group) Co. Ltd. The remaining authors declare that the research was conducted in the absence of any commercial or financial relationships that could be construed as a potential conflict of interest. The authors declare that this study received funding from Hongta Tobacco (Group) Co. Ltd. The funder had the following involvement in the study: sample collection, analysis, and field investigations.

## Publisher's note

All claims expressed in this article are solely those of the authors and do not necessarily represent those of their affiliated organizations, or those of the publisher, the editors and the reviewers. Any product that may be evaluated in this article, or claim that may be made by its manufacturer, is not guaranteed or endorsed by the publisher.
