# Erratum: Potential biocontrol efficiency of *Trichoderma* species against oomycete pathogens

**DOI:** 10.3389/fmicb.2022.1054303

**Published:** 2022-10-19

**Authors:** Frontiers Production Office

**Affiliations:** Frontiers Media SA, Lausanne, Switzerland

**Keywords:** oomycete disease, biological control, *Trichoderma* spp., control effect, antagonism

Due to a production error, an incorrect Funding statement was provided in which the funding number was omitted by mistake.

The Funding statement has now been corrected to:

“This study was supported financially by Hongta Tobacco (Group) Co. Ltd., Yuxi (2020YL04).”

Moreover, the old Funding statement has been moved to the Conflict of Interest statement, which now reads as follows:

“Authors JL, JW, JY, and YT were employed by Hongta Tobacco (Group) Co. Ltd.

The remaining authors declare that the research was conducted in the absence of any commercial or financial relationships that could be construed as a potential conflict of interest.

The authors declare that this study received funding from Hongta Tobacco (Group) Co. Ltd. The funder had the following involvement in the study: sample collection, analysis, and field investigations.”

The publisher apologizes for these mistakes. The original version of this article has been updated.

